# Supra-Uterine Pressure

**Published:** 1870-10

**Authors:** 


Supra-Uterine Pressure.—This is the name which Dr. J. H. Grant (*N. O. Med. Jour.*) gives to an expedient he has resorted to with success, as an aid in difficult labor. It consists in the application of strong, firm, and equable pressure over the whole region of the fundus uteri, at a stage of the labor when, with dilated, or dilatable os, with a normal presentation, and no obstacle to the labor, save the want of power to propel the fœtus through the parts, the labor does not progress. It is applicable to that class of cases in which the forceps and ergot are resorted to. He believes it may and ought to displace the use of these means altogether. In some of his cases a pressure was made of not less than forty pounds.

The hands of one or more assistants are to be spread out over the fundus uteri, and any amount of pressure necessary to enable the uterus to move forward its contents should be applied As soon as the pressure is commenced the pains augment in intensity, and return with great regularity. The pressure assists nature to do her work in her own way. The character of the pains differs from that induced by ergot; this drug excites the uterine fibra, inducing great irritability, and, if delivery does not speedily follow, may do much mischief. The pains from the pressure are natural; the uterus is not forced to expel its contents, but is assisted to do so. If the pressure is made to embrace a considerable portion of the body of the uterus, it has a tendency to support that organ and preserve it from rupture. Reason would teach that an organ like the womb cannot be ruptured by pressure in the longitudinal direction of its fibres. Supra-uterine pressure equalizes uterine action, and causes all parts of the organ to act simultaneously and regularly. Nor need we fear injury to the fœtus; the pressure is made on the fœtus as a whole, and not on any particular part. Obliquity of presentation, want of flexion, and rotation are deviations which commonly result from atonic action of the uterus. By physical assistance of the organ, bringing it to a point of vigorous action, these difficulties will be overcome.

This mode of practice promises to be very useful in placenta plævia. In these cases we can, in the early stage, hold the hæmorrhage in check with the tampon, till the os is dilated, when we can accelerate the advance of the child to any desired extent, and, as the after-birth separates from the cervix, the head of the child will form an efiection tampon, and restrain the bleeding till the birth of the child.

He usually prefers that, when this practice is resorted to, the parturient woman be sitting across the midwife's lap.—*N. E. Journal of Medicine.*

